# Identification, Bioactivity, and Productivity of Actinomycins from the Marine-Derived *Streptomyces heliomycini*

**DOI:** 10.3389/fmicb.2017.01147

**Published:** 2017-06-28

**Authors:** Dongyang Wang, Cong Wang, Pengyan Gui, Haishan Liu, Sameh M. H. Khalaf, Elsayed A. Elsayed, Mohammed A. M. Wadaan, Wael N. Hozzein, Weiming Zhu

**Affiliations:** ^1^Key Laboratory of Marine Drugs, MEC, School of Medicine and Pharmacy, Ocean University of ChinaQingdao, China; ^2^Bioproducts Research Chair, Zoology Department, College of Science, King Saud UniversityRiyadh, Saudi Arabi; ^3^Natural and Microbial Products Deptartment, National Research Centre, DokkiCairo, Egypt; ^4^Botany and Microbiology Department, Faculty of Science, Beni-Suef UniversityBeni-Suef, Egypt

**Keywords:** marine-derived actinobacteria, *Streptomyces heliomycini* WH1, actinomycins, cytotoxicity, antimicrobial activity

## Abstract

In the process of profiling the secondary metabolites of actinobacteria isolated from the Saudi coastal habitats for production of antibiotics and anti-cancer drugs, the cultures of strain WH1 that was identified as *Streptomyces heliomycini* exhibited strong antibacterial activity against *Staphylococcus aureus*. By means of MS and NMR techniques, the active compounds were characterized as actinomycins X_0β_, X_2_, and D, respectively. The research on the productivity of this strain for actinomycins revealed that the highest production of actinomycins X_0β_, X_2_, and D was reached in the medium MII within 5% salinity and pH 8.5. In this optimized condition, the fermentation titers of actinomycins X_0β_, X_2_, and D were 107.6 ± 4.2, 283.4 ± 75.3, and 458.0 ± 76.3 mg/L, respectively. All the three actinomycins X_0β_, X_2_, and D showed potent cytotoxicities against the MCF-7, K562, and A549 tumor cell lines, in which actinomycin X_2_ was the most active against the three tumor cell lines with the IC_50_ values of 0.8–1.8 nM. Both actinomycins X_2_ and D showed potent antibacterial activities against *S. aureus* and the methicillin-resistant *S. aureus, Bacillus subtilis*, and *B. cereus* and the actinomycin X_2_ was more potent.

## Introduction

The actinobacteria have been reported as the producers of two-thirds of the microbially-derived antibiotics known today (Newman et al., 2003). However, the rate for identification of novel compounds has decreased significantly from the widely explored normally terrestrial strains (Lam, 2006; Tiwari et al., 2015). Therefore, the discovery of new strains of actinobacteria may be the first and the key step to obtain novel compounds with bioactivity and subsequently to discover the natural product-based drugs. Increasing number of studies show that unusual and underexplored habitats, such as desert and marine ecosystems, are a rich source of novel actinobacteria with the capacity to produce new compounds with bioactivities (Bister et al., 2004; Bull and Stach, 2007; Fu et al., 2011, 2012, 2014; Wang et al., 2013).

**Figure 1:**
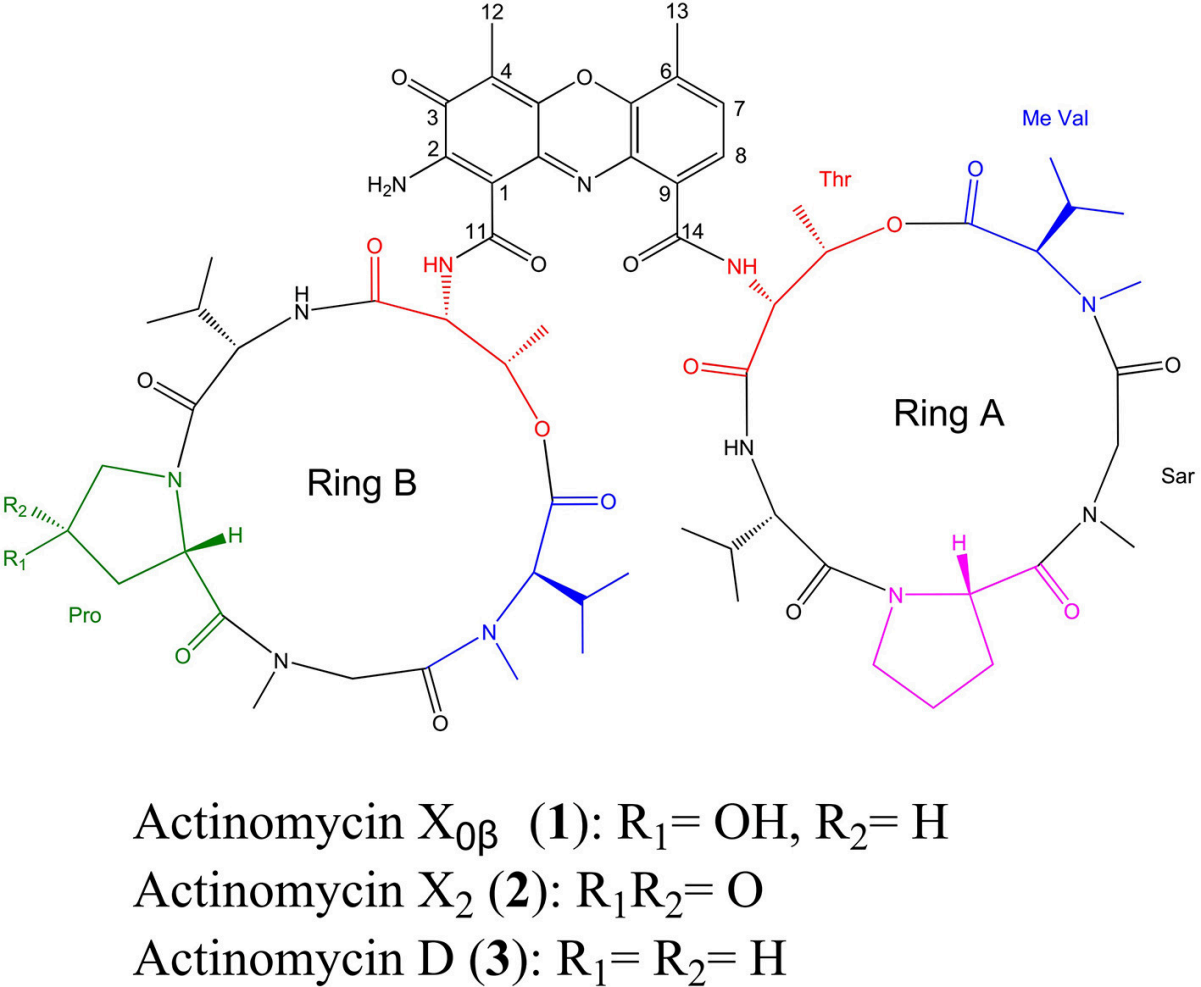
Chemical structures of Acts X_0β_ (**1**), X_2_ (**2**), and D (**3**) from *Streptomyces heliomycini* WH1.

Actinomycins are a class of chromopeptide antibiotics produced by *Streptomyces* sp., most of which share the same phenoxazinone chromophore. Actinomycin D (Act-D) is the most extensively studied example and is widely used as an anti-tumor drug for treatment of childhood rhabdomyosarcoma and Wilms' tumor, etc. The binding of Act-D to DNA is the basis for the antitumor activity (Koba and Konopa, 2005). This characteristic also makes Act D and 7-aminoactinomycin D as the useful tools for biological investigation (Chen Chiao et al., 1979). Act D also exhibit antiviral activity against coxsackievirus B3 (Saijets et al., 2003) and human immunodeficiency virus HIV-1 (Rill and Hecker, 1996), as well as the enzyme inhibitors against sereine proteinases (Betzel et al., 1993), acid phosphatase (Kapp and Okada, 1972), and tryptophan 2,3-dioxygenase (Killewich et al., 1975). However, its structurally related actinomycins (Acts), Act-X_2_ and Act-X_0β_, have not been well investigated for their medicinal properties due to the limits of the available amounts (Kurosawa et al., 2006). To discover new actinobacterial strains and optimize their cultural conditions to produce Acts, we carried out screenings of the marine-derived actinobacterial strains from the coastal habitats of Saudi Arabia. A producing strain of Acts, designated WH1, was identified as *Streptomyces heliomycini* whose products showed significant inhibition on the growth of *Staphyloccocus aureus*. A chemical study on the ethyl acetate (EtOAc) extract of the fermentation broth of *S. heliomycini* WH1 resulted in the isolation and identification of three Acts, Act-X_0β_ (**1**), Act-X_2_ (**2**), and Act-D (**3**) (Figure 1). All three Acts exhibited more potent cytotoxicities on the A549, MCF-7 and K562 tumor cell lines than adriamycin in which Act-X_2_ is the most active with the IC_50_ values of 0.8–1.8 nM and the lowest toxicity against the human embryo liver cell strand (L02 cells) with the values of the selective index (SI) of 5.2–12.2. Moreover, all the three Acts displayed more active or comparable antibacterial to ciprofloxacin hydrochloride against *Staphylococcus aureus* and the methicillin-resistant *S. aureus* (MRSA), *Bacillus subtilis*, and *Bacillus cereus* with MICs of 0.04–2.48 μM. In addition, the productivity on actinomycins of *S. heliomycini* WH1 under different cultural conditions were investigated.

## Materials and methods

### General experimental procedures

Silica gel (200–300 mesh) and on plates pre-coated with silica gel GF254 (10–40 μm) (Qingdao Marine Chemical Factory, Qingdao, China) were used in vacuum liquid chromatography (VLC) and thin layer chromatography (TLC), respectively. The optical rotation was measured by a Jasco P-1020 digital polarimeter. IR and UV spectra were recorded on a Nicolet Nexus 470 spectrophotometer using KBr discs and on a Hitachi UH5300 UV-Visible spectrophotometer, respectively. NMR spectra of Acts X_2_ and D were measured by a JEOL JNM-ECP 600 spectrometer while Act-X_0β_ was recorded on a Bruker Avance III 600 spectrometer and the chemical shifts were recorded as δ values using TMS as internal standard. Compounds were separately injected into the Q-TOF Ultima Global GAA076 LC mass spectrometer to obtained mass spectra. The cultures were analyzed over an analyzing YMC-ODS-A chromatographic column (4.6 × 250 mm, 5 μm) eluted with 80% H_2_O-MeOH (v/v) at a flow rate of 1 mL/min by a Shimadzu LC-6AD HPLC equipment and detected at λ_max_ 443 nm. The actinomycins were purified over a semi-preparative YMC-ODS-A chromatographic column (10 × 250 mm, 5 μm) eluted with 80% H_2_O-MeOH (v/v) at a flow of 4 mL/min by a Waters 1525 HPLC equipment. The RPMI 1640 powder with L-glutamine (Gln) and without NaHCO_3_ was from Life Technologies Corporation (USA). The ciprofloxacin hydrochloride was from J&K Scientific Ltd. (Beijing, China), and ketoconazole and itraconazole were from Energy Chemical (Shanghai, China). Phosphate Buffer Solution (PBS, 0.01 M, pH 7.2–7.4) and RPMI 1640 liquid medium for cell culture were from Beijing Solarbio Science & Technology Co., Ltd (Beijing, China).

### Bacterial material

The actinobacterial strain WH1 was isolated from a sandy soil sample collected at the coastal region of the Arabian Gulf at Jobail industrial city, in the Eastern Province of Saudi Arabia, and identified as *S. heliomycini* WH1 according to its phenotypic and phylogenetic characters (Figures S1, S2). The strain was deposited in our laboratories in 20% glycerol at −80°C. The working strain was prepared on ISP2 agar slants and stored at 4°C. The human pathogenic bacteria, *B. subtilis* (ATCC 6051), *Escherichia coli* (ATCC 11775), *Pseudomonas aeruginosa* (ATCC 10145), *Staphyloccocus aureus* (ATCC 6538) and MRSA (ATCC 43300), and the pathogenic fungi, *Candida albicans* (ATCC 10231) and *Candida glabrata* (ATCC 2001) were purchased from the Institute of microbiology, Chinese Academy of Sciences. The aquatic pathogenic bacteria, *B. cereus* (ATCC 14579), *Vibrio vulnificus* (ATCC 27562), and *Vibrio parahaemolyticus* (ATCC 17802) were purchased from Guangdong Institute of Microbiology (GIM). *Aspergillus fumigates* AF293 was given by Prof. Ling Lu, Nanjing Normal University, and *Vibrio alginolyticus, Vibrio splendidus*, and *Aeromonas hydrophila* were given by Prof. Xiangli Tian, Fisheries College of Ocean University of China.

### Isolation and identification of strain WH1

The collected sandy soil sample was air dried at room temperature (rt) for 7 days and then serially diluted up to 10^−4^ and inoculated in triplicates onto two selective media recommended for the isolation of actinobacteria, M1 (Mincer et al., 2002) and MM (Hozzein et al., 2008). After incubation at 28°C for 3 weeks, the isolate under study was picked and purified by streaking on the isolation medium twice each for 14 days at 28°C. The pure culture was maintained on slants at 4°C and preserved as a mixture of hyphae and spores in 20% glycerol at −80°C.

The purified isolate was characterized by its morphological characteristics (mycelia, cell morphology, and spore surface) by examining coverslip cultures on ISP2 agar plates grown at 28°C for 14 days by light and scanning electron (JEOL M6060) microscopes as described in the International Streptomyces Project (ISP) (Shirling and Gottlieb, 1966). The isomer type of the diaminopimelic acid in the cell wall and the whole-organism sugars were determined according to the standard methods of Hasegawa et al. (1983) and Staneck and Roberts (1974), respectively. The genomic DNA extraction, PCR amplification of the 16S rRNA gene, and sequencing of the PCR product were carried out as described before (Hozzein and Goodfellow, 2007). The obtained sequence was deposited in Genbank (accession No. AB184712) and compared with available 16S rRNA gene sequences of validly published species from the EzTaxon-e server (http://eztaxon-e.ezbiocloud.net/; Kim et al., 2012).

### Cultural media

The isolation medium was MM agar medium containing 0.05% glucose, 0.05% yeast extract, 0.05% MgSO_4_·7H_2_O, 0.05% NaCl, 0.1% K_2_HPO_4_, 1.8% agar, and 1 L seawater, pH 7.5. The working strain was prepared on ISP2 agar slants composed of 0.4% glucose, 1% malt extract, 0.4% yeast extract, 1.8% agar, and 1 L 50% seawater, pH 7.5. The MM liquid medium, soybean meal medium (2% soybean meal and 1 L seawater, pH 8.0), and the MI–MIV media were used to investigate the productivity of WH1 for actinomycins. MI–MIV media contained 2.0% yeast extract, 0.15% KH_2_PO_4_, 0.05% MgSO_4_·7H_2_O and 1 L seawater (pH 8.0), 2.0% soybean meal, 0.15% KH_2_PO_4_, 0.05% MgSO_4_·7H_2_O and 1 L seawater (pH 8.0), 2.25% soluble starch, 0.5% yeast extract, 0.15% KH_2_PO_4_, 0.05% MgSO_4_·7H_2_O and 1 L seawater (pH 8.0), and 2.25% glucose, 0.5% yeast extract, 0.15% KH_2_PO_4_, 0.05% MgSO_4_·7H_2_O and 1 L seawater (pH 8.0), respectively. The LB agar medium consisted of 1% tryptone, 0.5% yeast extract, 0.5% NaCl, 1.8% agar, and 1 L tap water (pH 7.4), while the YPD agar medium consisted of 1% yeast extract, 2% peptone, 2% glucose, 1.8% agar, and 1 L tap water (pH 7.0). The 2216E agar medium was prepared by 1% peptone, 0.5% yeast extract powder, 1.8% agar, and 1 L seawater (pH 7.8). The PDA agar medium contained 20% potato, 2% glucose, 1.8% agar, and 1 L tap water.

### Fermentation and extraction

*S. heliomycini* WH1 was fermented in ten 500-mL Erlenmeyer flasks each containing 150 mL MM liquid medium and was shaken for 10 days at 28°C and 180 rpm. The fermentation broth was extracted three times each with 1,500 mL EtOAc. The EtOAc phase was combined and evaporated to dryness under reduced pressure by a rotary evaporator to give the EtOAc extracts (0.5 g). *S. heliomycini* WH1 was also cultured in different media under different pH and salinity. The chemo-diversity of the EtOAc extracts was investigated by high performance liquid chromatography (HPLC, Figure S3).

### Purification and identification of the acts

The EtOAc extract (0.5 g) was separated into five fractions on a VLC silica gel column using a step gradient elution with 100:0, 50:1, 30:1, 10:1, and 0:100 (v/v) of CH_2_Cl_2_-MeOH. Then the fractions 2–4 containing actinomycins were combined and purified by semi-preparative HPLC using YMC-pack semi-preparative chromatographic column (ODS-A) eluted with 80% H_2_O-MeOH (v/v) at a flow rate of 4 mL/min to give Act-X_0β_ (**1**) (1.0 mg, t_R_ 9.6 min), Act-X_2_ (**2**) (3.7 mg, t_R_ 11.2 min) and Act-D (**3**) (6.6 mg, t_R_ 13.0 min). The isolation yields of Acts X_0β_, X_2_, and D were 0.7, 2.5, and 4.4 mg/L, respectively.

### Sample preparation for analysis of the acts production

The strain WH1 was fermented with three parallels for 10 days at 180 rpm and 28°C in a 500 mL Erlenmeyer flask containing 150 ml of MM liquid, soybean meal, and MI–MIV media, and the pH value was adjusted to a certain value before sterilization. The initial pH values were adjusted by 20% hydrochloric acid (HCl) or 4% sodium hydroxide (NaOH). The 0, 3, 5, and 7% salinity were prepared by tap water, seawater, and sea water supplemented with NaCl, respectively. Each experiment was carried out in three parallel. The fermentation broths were extracted thrice with EtOAc (each 250 mL), and concentrated to dryness *in vacuo* to give the extracts for HPLC analysis.

### Analysis of the acts production

The production of Acts was estimated by establishing the standard curve between the HPLC peak areas and the concentrations of Acts. The standard curve was established using standard solutions from 0.1 to 10 μg/mL on a YMC-pack C18 analytical column with 1 mL/min of flow rate and detection at λ_max_ 443 nm. Linear curves and their fitting equations were established using Origin 9.0. The production of Acts was calculated according to the fitting equations.

### Cytotoxic assay of the acts

The cytotoxicities on three human cancer cell lines, nonsmall cell lung cancer cell line (A549), breast cancer cell line (MCF-7), and myelogenous leukemia cell line (K562), along with human embryo liver L02 cell line were assayed. The method for A549, MCF-7 and L02 was the 3-(4,5-Dimethylthiazol-2-yl)-2,5-diphenyltetrazolium bromide (MTT) (Mosmann, 1983), while the one for K562 was the cell counting Kit-8 (CCK-8) (Tominaga et al., 1999). A549, MCF-7, K562, and L02 cell lines were cultured for 3–5 d in the RPMI-1640 liquid medium supplemented with 10% FBS under a humidified atmosphere with 5% CO_2_ and 95% air at 37°C. Then 100 μL of the cell suspensions with a density of 3 × 10^5^ cells mL^−1^ was plated in the 96-well microtiter plates and incubated for 12 h. The 200 μM testing DMSO solutions of the samples were diluted into 12.5 and then to 0.012 μM by the continuous 2-fold dilution method with RPMI-1640 medium. Then, the obtained test solutions (100 μL) were added into above wells each containing 100 μL cell suspension and further incubated for 72 h. The 20 μL 0.5% MTT solution (in PBS) was added to each well containing A549, MCF-7, and L02 cell lines and further incubated for 4 h. The culture broth was then gently pipetted and the DMSO (150 μL) was added to dissolve the formed formazan crystals. Absorbance of the solution was determined on a Spectra Max Plus plate reader at 570 nm. The CCK-8 solution was added to each well containing K562 cell and further incubated for 6 h, absorbance was determined on a Spectra Max Plus plate reader at 450 nm. The inhibition rates were calculated as ((A_blankcontrol_ − A_sample_)/A_blankcontrol_ × 100%). The half-maximal inhibitory concentration (IC_50_) is defined as the concentration within 50% inhibition. Adriamycin was used as the positive control with the IC_50_ values of 1.30, 0.30, and 1.00 μM for A549, K562, and MCF-7, and the CC_50_ value of 0.40 μM for L02 cell lines, respectively. The selective index (SI) is defined as the value of CC_50_/IC_50_.

### Antimicrobial assay of the acts

The antimicrobial activities against human pathogenic bacteria (*B. subtilis, E. coli, P. aeruginosa, S. aureus*, and MRSA) and pathogenic fungi (*C. albicans, C. glabrata, Aspergillus fumigatus* AF293) and aquatic pathogenic bacteria (*A. hydrophila, B. cereus, V. alginolyticus, V. parahaemolyticus, V. splendidus*, and *V. vulnificus*) were evaluated using the filter paper disc method. The pathogenic strains were cultivated in LB agar plates at 37°C for bacteria and in YPD agar plates at 37°C for fungi. The testing methanol (MeOH) solutions (1 mg/mL) of the samples and positive control (ciprofloxacin hydrochloride for bacteria, and ketoconazole for *C. albicans* and *C. glabrata*) were diluted into 500–1.95 μg/mL by the continuous 2-fold dilution method with MeOH. Then 10 μL of the testing solutions were separately added to the paper disc (5 mm diameter). After evaporation to dryness, the drug paper discs were added into the cultural plates of the pathogenic microorganisms and incubated at 28°C for 12 h. Inhibition zones were then recorded as mm in diameter. The samples were first tested for their inhibitory zone diameters (IZDs) at the concentration of 1 mg/mL. Only those active samples with IZDs ≥14 mm were tested for their minimum inhibitory concentration (MIC) by 2-fold dilution method (Fu et al., 2013). The drug solutions of the three actinomycins, extracts and ciprofloxacin hydrochloride (positive control) were respectively prepared by a serial 2-fold dilution method from 100 to 0.049 μg/mL with the LB liquid medium for *S. aureus* and *B. subtilis* and 2216E liquid medium for *B. cereus*. The pathogenic bacterial colony with 24 h old grown on the LB (*S. aureus* and *B. subtilis*) or 2216E (*B. cereus*) agar plates were transferred into a 50-mL tube containing 30 mL fresh corresponding liquid media and incubated for 12 h at 28°C and 180 rpm. The final bacterial suspension was adjusted to the density of 5 × 10^5^ CFU/mL with fresh corresponding liquid media and was added into the 96-well plates. Each well contains 100 μL bacterial suspension and 100 μL of the testing solution. The medium (100 μL) equipped with 100 μL bacterial suspension was used as the corresponding negative controls and the medium (200 μL) was used as blank controls. Each experiment was carried out in three parallel wells. All plates were stationary incubated for 15 h at 37°C. The minimal inhibitory concentration (MIC) was the lowest drug concentration at which no bacteria were grown, that is, the wells were more transparent than the negative control examined by eyes. In addition, the antifungal activity against *A. fumigatus* AF293 was also assayed. 3-(N-morpholino) propansulfonic acid (MOPS, 6.906 g) and glucose (4 g) were dissolved in 65 mL of deionized water at 60°C and then cooled to rt. And then 2.08 g RPMI 1640 powder with L-Gln and without NaHCO_3_ was added to the solution. The pH was adjusted to 7.0 with 5.0% NaOH and the solution volume was adjusted to 90 mL by adding deionized water. The *A. fumigatus* was grown on PDA at 28°C for a week and the mature spores were suspended in 0.9% saline and the density was adjusted to 2 × 10^4^ CFU/mL with above fresh-prepared RPMI 1640 medium. The drug solution was prepared by dissolving extracts (50 mg/mL) or compounds (10 mg/mL) in DMSO and then diluted to 100-fold with the sterile water so that the final concentration of DMSO was less than 1%. One hundred microliters of the drug solution was added into 96-well plates (Costar 3599) that each well contains 100 μL *A. fumigatus* AF293 suspension within the density of 2 × 10^4^ CFU/mL. The 96-well plates were incubated in a wet box at 28°C for 4–7 days. Itraconazole, *A. fumigatus* AF293 suspension without drugs and the fresh-prepared RPMI 1640 medium were used as the positive control, growth control, and the negative control, respectively. Each experiment was set in three parallels. The MIC was the lowest drug concentration at which no fungal growth was observed compared to the growth control.

## Results

### Identification of strain WH1

The observed morphological features of strain WH1 showed that it produced extensively branched hyphae bearing long spore chains with smooth surfaces (Figure S1). The cell walls of the strain WH1 contained LL-DAP as the characteristic amino acid of the peptidoglycan and its whole-organism sugar patter have glucose, galactose, and mannose as the characteristic sugars. These characters indicated that WH1 belongs to genus Streptomyces. The taxonomic position of WH1 and its affiliation to genus Streptomyces was confirmed by the analysis of the 16S rRNA gene sequence against the available validly published species. The sequence analysis using the EzTaxon-e server showed that strain WH1 had 100% similarity to *S. heliomycini* NCBR 15899(T). These results indicated that the strain WH1 under study is a strain of *S. heliomycini*.

### The identification of acts X_0β_, X_2_, and D

HPLC analysis revealed that there were three peaks with the typical UV absorption of Acts at λ_max_ 203, 225, and 443 nm in the cultures of *S. heliomycini* WH1 (Wang et al., 2014) (Figure S3). Chemical investigations resulted in the isolation of the three Acts, compounds **1**–**3**, by VLC and semi-preparative HPLC. By means of specific rotation, MS and NMR data, their structures were identified. The ^13^C NMR spectra of compounds **1**–**3** showed the characteristic skeleton resonances of Acts at δ 34–40 for N-CH_3_ (× 4) and δ 165–175 for N-C=O (× 12), further indicating the nature of the Acts of compounds **1**–**3**. All the ^13^C NMR spectra of compounds **1**–**3** showed 62 carbon signals (Figures S5, S8, S11) and the ESIMS of compounds **1**–**3** showed molecular peaks at *m/z* 1272.06 [M+H]^+^ (Figure S6), 1270.03 [M+H]^+^ (Figure S9), and 1256.05[M+H]^+^ (Figure S12). Their molecular formulas were further determined as C_62_H_86_O_17_N_12_, C_62_H_84_N_12_O_17_, and C_62_H_86_N_12_O_16_ from the HRESIMS peaks at *m/z* 1271.6305[M+H]^+^ (Figure S6), 1269.6155[M+H]^+^ (Figure S9), and 1255.6365[M+H]^+^ (Figure S12), respectively. The differences of ^13^C NMR spectra of compounds **1**–**3** are that a methylene carbon signal (δ_C_ 23.0) in **3** is replaced by an oxygenated methine carbon signal (δ_C_ 70.0) in **1** and a carbonyl signal (δ_C_208.8) in **2**, respectively. These data suggested that compounds **1**–**3** might be corresponding to Acts X_0β_, X_2_, and D. The consistence of NMR (Figures S4, S5, S7, S8, S10, S11, Tables 1–3) and [α]_D_ with those reported further supported Acts **1**–**3** were Act-X_0β_ (**1**) (Lifferth et al., 1999), Act-X_2_ (**2**) (Lifferth et al., 1999) and Act-D (**3**) (Wang et al., 2014), respectively (Figure 1).

**Table 1:** ^1^H (600 MHz) and ^13^C (150 MHz) NMR Date of Act-X_0β_ (**1**) in CDCl_3_^a^.

	**Ring-A**			**Ring-B**		
	**Position**	**δ_C_, type**	**δ_H_ (*J* in Hz)**	**Position**	**δ_C_, type**	**δ_H_ (*J* in Hz)**
Thr	1	168.8, C		1	169.0, C	
	2	55.5, CH	4.81 (dd, 7.0, 2.5)	2	54.9, CH	4.49 (dd, 6.5, 2.8)
	3	74.7, CH	5.24 (m)	3	75.4, CH	5.24 (m)
	4	17.7, CH_3_	1.28 (d, 6.3)	4	17.8, CH_3_	1.24 (d, 6.0)
	NH		7.44 (d, 6.9)	NH		7.50 (d, 6.4)
Val	1	173.5, C		1	173.3, C	
	2	58.9, CH	3.57 (dd, 10.2, 3.5)	2	58.1, CH	3.55 (dd, 8.9, 4.3)
	3	32.0, CH	2.16 (m)	3	31.9, CH	2.12 (m)
	4	19.2, CH_3_	0.75 (d, 2.7)	4	19.2, CH_3_	0.74 (d, 2.7)
	5	19.2, CH_3_	0.96 (d, 7.4)	5	19.0, CH_3_	0.94 (d, 1.6)
	NH		8.17 (d, 6.2)	NH		7.91 (d, 5.5)
Pro	1	173.2, C		1	173.0, C	
	2	57.0, CH	5.97 (d, 9.2)	2	56.5, CH	6.05 (d, 9.6)
	3	31.2, CH_2_	1.84 (m), 2.78 (m)	3	31.4, CH_2_	4.13 (m), 3.95 (m)
	4	23.0, CH_2_	2.06 (m), 2.26 (m)	4	70.0, CH	4.70 (m)
	5	47.6, CH_2_	3.85 (m), 3.73 (m)	5	54.7, CH_2_	3.56 (m), 3.08 (m)
Sar	1	166.5, C		1	166.5, C	
	2	51.3, CH_2_	4.72 (d, 17.4), 3.63 (d, 17.4)	2	51.5, CH_2_	4.55 (d, 17.4), 3.59 (d, 17.4)
MeVal	NMe	35.0, CH_3_	2.87 (s)	NMe	35.1, CH_3_	2.87 (s)
	1	167.6, C		1	167.7, C	
	2	71.3, CH	2.71 (d, 9.3)	2	71.4, CH	2.67 (d, 9.4)
	3	27.0, CH	2.78 (m)	3	27.0, CH	2.64 (m)
	4	21.7, CH_3_	0.96 (m)	4	21.7, CH_3_	0.94 (m)
	5	19.4, CH_3_	0.75 (d, 2.7)	5	19.2, CH_3_	0.74 (d, 3.1)
	NMe	39.5, CH_3_	2.95 (s)	NMe	39.3, CH_3_	2.94 (s)
chromophore	1	102.7, C				
	2	147.1, C				
	3	179.1, C				
	4	113.6, C				
	4a	145.0, C				
	5a	140.7, C				
	6	128.5, C				
	7	130.4, CH	7.35 (d, 7.8)			
	8	126.2, CH	7.64 (d, 7.8)			
	9	131.3, C				
	9a	129.6, C				
	10a	146.1, C				
	11	166.2, C				
	12	7.9, CH_3_	2.22 (s)			
	13	15.3, CH_3_	2.54 (s)			
	14	173.0, C				

a *Thr, Threonine; Val, Valine; Pro, Proline; Sar, Sarcosine*.

**Table 2:** ^1^H (500 MHz) and ^13^C (125 MHz) NMR Date of Act-X_2_ (**2**) in CDCl_3_^a^.

	**Ring-A**			**Ring-B**		
	**Position**	**δ_C_, type**	**δ_H_ (*J* in Hz)**	**Position**	**δ_C_, type**	**δ_H_ (*J* in Hz)**
Thr	1	168.7, C		1	168.8, C	
	2	55.0, CH	4.55 (m)	2	54.8, CH	4.48 (m)
	3	74.7, CH	5.15 (m)	3	74.6, CH	5.24 (m)
	4	17.2, CH_3_	1.14 (d, 6.4)	4	17.7, CH_3_	1.26 (d, 7.3)
	NH		7.17 (d, 7.2)	NH		7.67 (d, 6.1)
Val	1	173.5, C		1	174.0, C	
	2	58.5, CH	3.57 (dd, 9.3, 6.3)	2	57.2, CH	3.70 (dd, 10.4, 6.3)
	3	31.9, CH	2.10 (m)	3	31.7, CH	2.09 (m)
	4	18.9, CH_3_	0.90 (d, 6.8)	4	18.8, CH_3_	0.89 (d, 6.8)
	5	19.2, CH_3_	1.12 (d,6.8)	5	19.2, CH_3_	1.15 (d, 6.8)
	NH		7.68 (d, 6.2)	NH		8.19 (d, 5.9)
Pro	1	173.1, C		1	172.7, C	
	2	56.4, CH	5.95 (d, 8.9)	2	54.3, CH	6.55 (d, 11.0)
	3	31.0, CH_2_	1.84 (m), 2.75 (m)	3	41.9, CH_2_	3.85 (m), 2.33 (m)
	4	23.0, CH	2.24 (m)	4	208.8, C	
	5	47.4, CH_2_	3.91 (m), 3.74 (m)	5	52.9, CH_2_	4.55 (m), 3.89 (m)
Sar	1	166.0, C		1	165.9, C	
	2	51.3, CH_2_	4.72 (d, 17.3), 3.62 (d, 17.3)	2	51.3, CH_2_	4.57 (d, 17.3), 3.62 (d, 17.3)
MeVal	NMe	34.8, CH_3_	2.88 (s)	NMe	34.9, CH_3_	2.89 (s)
	1	166.5, C		1	166.3, C	
	2	71.3, CH	2.68 (m)	2	71.5 CH	2.68 (m)
	3	26.9, CH	2.68 (m)	3	27.0, CH	2.68 (m)
	4	21.6, CH_3_	0.94 (d, 5.8)	4	21.7, CH_3_	0.97 (d, 5.5)
	5	19.1, CH_3_	0.74 (d, 5.8)	5	19.1, CH_3_	0.73 (d, 5.5)
	NMe	31.7, CH_3_	2.92 (s)	NMe	31.9, CH_3_	2.93 (s)
chromophore	1	101.7, C				
	2	147.4, C				
	3	179.0, C				
	4	113.6, C				
	4a	145.0, C				
	5a	140.5, C				
	6	127.8, C				
	7	130.3, CH	7.35 (d, 7.8)			
	8	126.2, CH	7.60 (d, 7.8)			
	9	132.1, C				
	9a	129.1, C				
	10a	145.9, C				
	11	167.4, C				
	12	7.8, CH_3_	2.24 (s)			
	13	15.1, CH_3_	2.55 (s)			
	14	172.7, C				

a *Thr, Threonine; Val, Valine; Pro, Proline; Sar, Sarcosine*.

**Table 3:** ^1^H (500 MHz) and ^13^C (125 MHz) NMR Date of Act-D (**3**) in CDCl_3_^a^.

	**Ring-A**			**Ring-B**		
	**Position**	**δ_C_, type**	**δ_H_ (*J* in Hz)**	**Position**	**δ_C_, type**	**δ_H_ (*J* in Hz)**
Thr	1	169.0, C		1	168.5, C	
	2	55.4, CH	4.60 (d, 5.1)	2	55.0, CH	4.48 (d, 5.2)
	3	75.2, CH	5.20 (d, 5.1)	3	75.1, CH	5.16 (d, 5.6)
	4	17.5, CH_3_	1.26 (s)	4	18.0, CH_3_	1.26 (s)
	NH		7.19 (d, 6.4)	NH		7.81 (d, 5.8)
Val	1	173.8, C		1	173.5, C	
	2	59.0 CH	3.54 (m)	2	58.8, CH	3.55 (m)
	3	31.9, CH	2.16 (m)	3	31.6, CH	2.08 (m)
	4	19.4, CH_3_	1.12 (d, 5.7)	4	19.2, CH_3_	1.12 (d, 5.7)
	5	19.2, CH_3_	0.9 (d, 6.7)	5	19.1, CH_3_	0.89 (d, 6.7)
	NH		8.09 (d, 5.3)	NH		7.94 (d, 5.7)
Pro	1	173.4 C		1	173.4, C	
	2	56.4, CH	6.02 (d, 9.0)	2	56.3, CH	5.98 (d, 9.0)
	3	31.1, CH_2_	1.88 (m), 2.67 (m)	3	31.4, CH_2_	1.87 (m), 2.67 (m)
	4	23.1, CH	2.17 (m), 2.25 (m)	4	23.0, CH_2_	2.15 (m), 2.25 (m)
	5	47.7, CH_2_	3.72 (m)	5	47.4, CH_2_	3.82 (m)
Sar	1	166.4, C		1	166.1, C	
	2	51.5, CH_2_	4.76 (d, 17.6), 3.61 (d, 17.6)	2	51.5, CH_2_	4.70 (d, 17.4), 3.64 (d, 17.5)
MeVal	NMe	35.0, CH_3_	2.88 (s)	NMe	35.0, CH_3_	2.88 (s)
	1	167.8, C		1	167.7, C	
	2	71.6, CH	2.67 (m)	2	71.4, CH	2.67 (m)
	3	27.0, CH	2.67 (m)	3	27.0, CH	2.67 (m)
	4	21.8, CH_3_	0.96 (d, 7.4)	4	21.7, CH_3_	0.95 (d, 6.6)
	5	19.4, CH_3_	0.75 (s)	5	19.2, CH_3_	0.75 (s)
	NMe	39.4, CH_3_	2.90 (s)	NMe	39.3, CH_3_	2.94 (s)
chromophore	1	101.8, C				
	2	147.8, C				
	3	179.2, C				
	4	113.6, C				
	4a	145.2, C				
	5a	140.6, C				
	6	127.7, C				
	7	130.4, CH	7.37 (d, 7.7)			
	8	126.0, CH	7.64 (d, 7.7)			
	9	132.7, C				
	9a	129.2, C				
	10a	146.0, C				
	11	169.0, C				
	12	7.9, CH_3_	2.25 (s)			
	13	15.2, CH_3_	2.56 (s)			
	14	166.6.0, C				

a *Thr, Threonine; Val, Valine; Pro, Proline; Sar, Sarcosine*.

### The standard curves for analysis of the productions of acts

**Figure 2:**
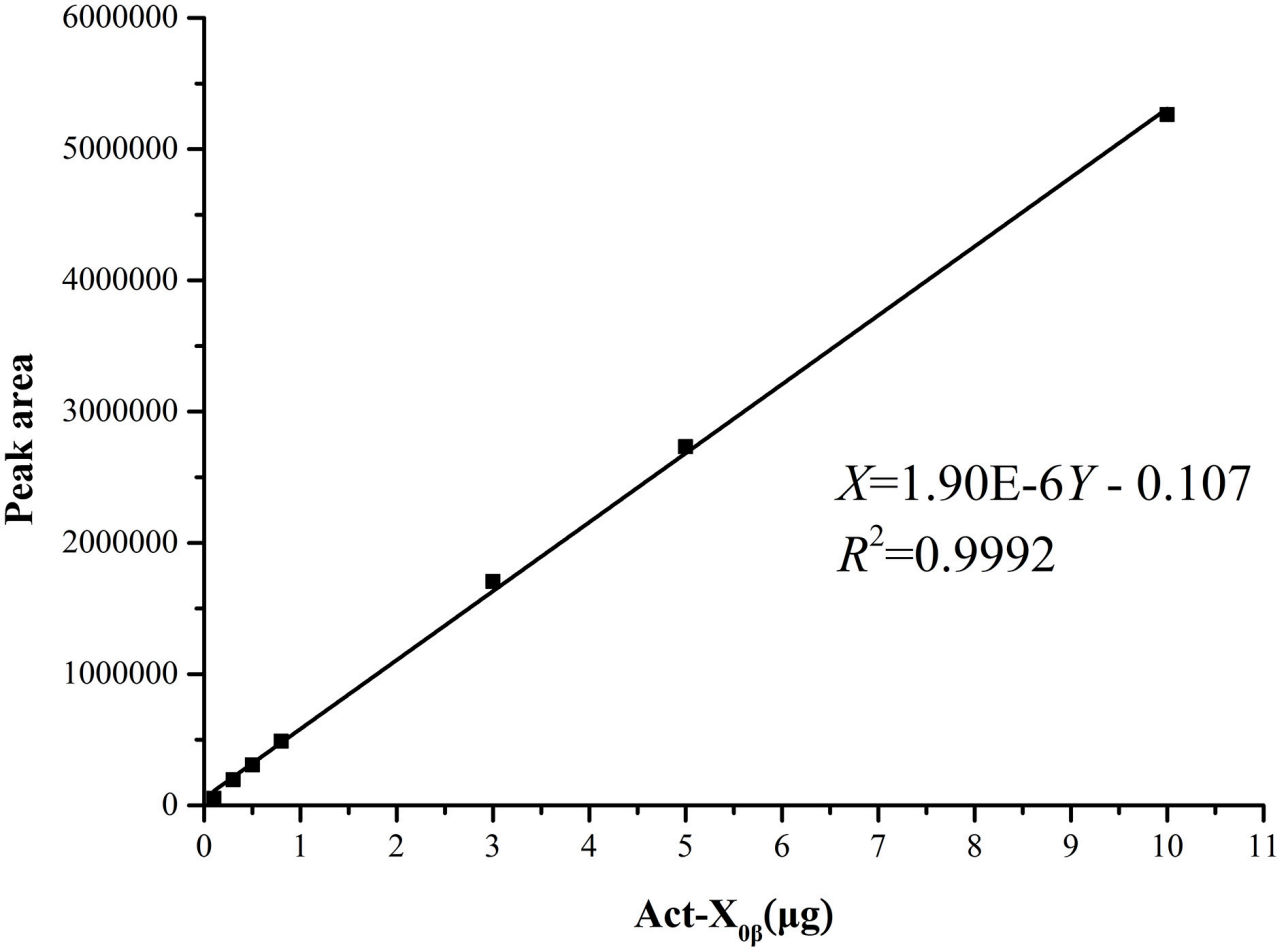
Standard carve of Act-X_0β._

**Figure 3:**
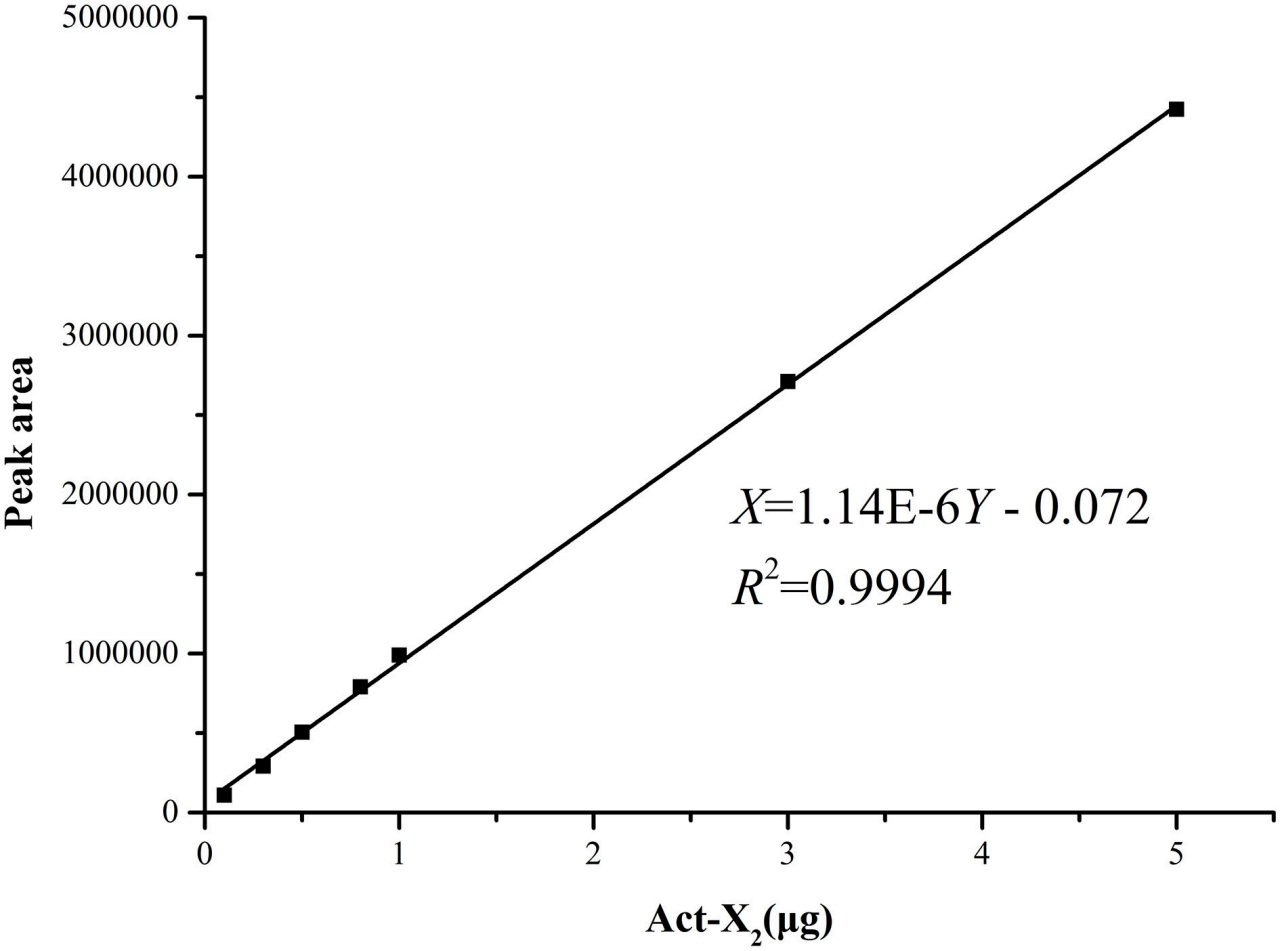
Standard carve of Act-X_2._

**Figure 4:**
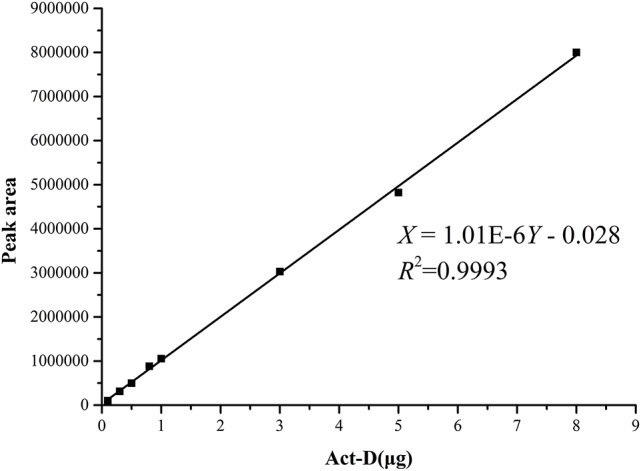
Standard carve of Act-D.

The standard curves of Acts X_0β_, X_2_, and D were established by means of HPLC-UV. The liner regression equations for ActsX_0β_, X_2_, and D were respectively obtained as X = 1.90E–6Y – 0.107 (*R*^2^ = 0.9992) (Figure 2), X = 1.14E–6Y – 0.072 (*R*^2^ = 0.9994) (Figure 3), and X = 1.01E–6Y – 0.028 (*R*^2^ = 0.9993) (Figure 4), where Y is the weight of Acts (μg) and X is the peak area. All curves showed good linear relationships that could be used to estimate the production of the Acts from the corresponding HPLC peaks' areas.

### The effects of pH, salinity, and media on productivity of acts from *S. heliomycini* WH1

**Figure 5:**
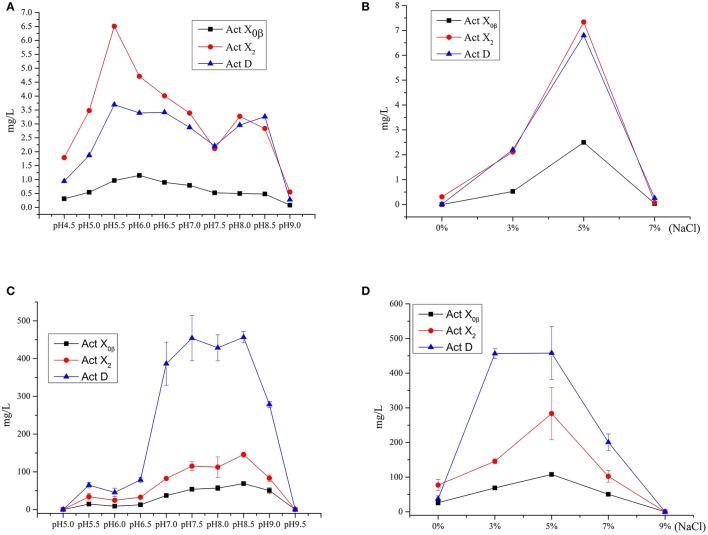
The effect of the initial pH values and salinity on the productions of Acts X_0β_, X_2_ and D. **(A)** The effect of the initial pH values on the productions of Acts X_0β_, X_2_ and D in the liquid medium MM. **(B)** The effect of the salinity on the productions of Acts X_0β_, X_2_ and D in the liquid medium MM. **(C)** The effect of the initial pH values on the productions of Acts X_0β_, X_2_ and D in the liquid medium MII. **(D)** The effect of the salinity on the productions of Acts X_0β_, X_2_ and D in the liquid medium MII.

The effect of the initial pH values on the production was studied in the MM liquid medium with 3% salinity (natural seawater), whose initial pH was increased to 9.0 from 4.5 at an interval of 0.5. The results (Figure 5A, Table S1) showed that the production of Acts X_0β_, X_2_, and D reached the highest at pH 6.0, 5.5, and 5.5 in the MM medium, whose yields were 1.2, 6.5 and 3.7 mg/L, respectively. This indicated that 5.5 is the most suitable initial pH value for producing both Acts D and X_2_ in MM medium, and is also suitable for producing Act-X_0β_ that was 1.0 mg/L only after the production at pH 6.0. The reason is that all the components in the MM liquid medium were completely dissolved at pH 5.5. Only under this pH value, the MM liquid medium is clear and transparent that is easy to be used by microorganisms.

The effect of the salinity on the Acts production was studied in the MM liquid medium at pH 5.5. The salinity of the medium was designed as 0, 3, 5, and 7%, respectively. The results (Figure 5B, Table S2) showed that the highest production of Acts X_0β_, X_2_, and D was under 5% salinity with the yields of 2.5, 7.3, and 6.8 mg/L, respectively, while the corresponding production under 3% salinity was 0.5, 2.1, and 2.2 mg/L, respectively. However, the Acts fermentation titers under these conditions in MM liquid medium are too low to be satisfactory. Therefore, the other media were adopted to improve the productions of the Acts.

The study on the productivity of Acts in the soybean meal and MI–MIV liquid media (Table 4) showed that the highest production of all the Acts X_0β_, X_2_, and D was in the MII liquid medium with the yields of 56.8 ± 6.8, 112.4 ± 27.2, and 428.5 ± 34.5 mg/L, respectively. The results indicated that the soybean meal supplemented with the minor elements Mg, K, P, and S, that is MII liquid medium, is favorable for the production performance of actinomycins by strain *S. heliomycini* WH1. Therefore, the productivity of Acts was further optimized in the liquid medium MII by investigating the effects of pH values and salinity. The results showed that initial pH 8.5 was the most suitable pH value for production of all the Acts X_0β_, X_2_, and D in the medium MII with the yields of 68.8 ± 1.2, 145.7 ± 6.8, and 456.5 ± 14.7 mg/L (Figure 5C, Table S3) at 3% salinity, respectively. On the basis of initial pH 8.5, the highest productivity of all the Acts X_0β_, X_2_, and D was obtained in the liquid medium MII with 5% salinity which reached to 107.6 ± 4.2, 283.4 ± 75.3, and 458.0 ± 76.3 mg/L (Figure 5D, Table S4), respectively.

**Table 4:** The productions of Acts X_0β_, X_2_, and D in different media (mg/L).

**Medium**	**Act-X_0β_**	**Act-X_2_**	**Act-D**
MM	0.7 ± 0.02	2.5 ± 0.2	4.4 ± 0.2
MI	36.0 ± 3.4	34.2 ± 3.5	173.4 ± 20.5
MII	56.8 ± 6.8	112.4 ± 27.2	428.5 ± 34.5
MIII	38.7 ± 1.7	90.7 ± 14.1	162.0 ± 10.3
MIV	53.8 ± 5.4	219.7 ± 23.8	269.7 ± 40.3
Soybean meal	61.5 ± 15.7	121.6 ± 32.0	330.8 ± 64.2

### The bioactivities of acts from *S. heliomycini* WH1

The cytotoxicities of Acts X_0β_, X_2_, and D on the A549, MCF-7, and K562 and L02 cell lines were examined. The results indicated that all the three Acts exhibited strong cytotoxicities on the three tumor cell lines and the one normal cell line with the IC_50_ and CC_50_ values of 0.8–157.4 nM (Table 5). Among them, Act-X_2_ displayed the strongest activities on the three human tumor cell lines, A549, MCF-7 and K562, and the lowest toxicity on the human normal embryo liver L02 cell line. Thus, Act-X_2_ showed the highest selective index (SI) for the three tested tumor cell lines (10.3, 12.2, and 5.2, respectively), indicating a potential of Act-X_2_ as a drug candidate for treatment of human cancers. As far as we known, there were no reports on the cytotoxicity of Acts X_0β_ and X_2_ on the A549, MCF-7 and K562 tumor cells.

**Table 5:** The cytotoxicity and selective index (SI) of Acts X_0β_, X_2_, and D.

**Cell lines**	**Act-X**_**0β**_		**Act-X**_**2**_		**Act-D**		**Adriamycin**	
	**IC_50_ (nM)**	**SI**	**IC_50_ (nM)**	**SI**	**IC_50_ (nM)**	**SI**	**IC_50_ (nM)**	**SI**
A549	63.8 ± 2.9	2.5 ± 0.3	0.9 ± 0.1	10.4 ± 1.9	3.4 ± 0.1	3.9 ± 0.3	1,300	0.3
MCF-7	85.2 ± 12.2	1.9 ± 0.4	0.8 ± 0.1	12.2 ± 1.8	2.6 ± 0.1	4.9 ± 0.4	1,000	0.4
K562	128.3 ± 6.1	1.2 ± 0.2	1.8 ± 0.1	5.2 ± 0.5	3.3 ± 0.3	4.0 ± 0.5	300	1.3
L02 (CC_50_)	157.4 ± 14.7		9.3 ± 0.7		13.0 ± 0.5		400	

The antimicrobial activities against the human and aquatic pathogenic microbes, *A. hydrophila, B. subtilis, B. cereus, E. coli, P. aeruginosa, S. aureus*, MRSA, *V. vulnificus, V. alginolyticus, V. parahaemolyticus, V. splendidus, C. albicans, C. glabrata, A. fumigatus* AF293, and were evaluated. The results indicated that both Act-X_2_ and Act-D showed comparable or stronger antimicrobial activities against *S. aureus*, MRSA, *B. subtilis*, and *B. cereus* to ciprofloxacin hydrochloride (a positive control, MIC 0.1–12.5 μM) with MIC values of 0.04–0.15 μM (Table 6), while Act-X_0β_ displayed very weak inhibitions on *S. aureus* and *B. subtilis* (MIC 0.3–2.5 μM). All the three Acts were not active against the tested pathogenic fungi and other bacteria at the concentration of 1 mg/mL. Except for the antibacterial activities of Act-X_2_ on the *S. aureus* and *B. cereus* (Xiong et al., 2008) and Act-D on the *S. aureus* (Bian et al., 2003), there were no other reports on the antibacterial activities of Acts D, X_2_ and X_0β_ against MRSA and *B. subtilis*, the Act-X_0β_ against *S. aureus* and *B. cereus*, as well as the Act-D against *B. cereus*. And the Acts D and X_2_ were more active. These results revealed the potential use of Act-X_2_ and Act-D in the treatment of infectious diseases caused by *S. aureus, B. subtilis*, and *B. cereus*, especially by MRSA.

**Table 6:** The MIC values (μM) of Acts X_0β_, X_2_, and D on the pathogenic bacteria.

**Bacteria**	**Act-X_0β_**	**Act-X_2_**	**Act-D**	**Ciprofloxacin hydrochloride**
*S. aureus*	0.31	0.15	0.31	0.53
MRSA	2.48	0.15	0.31	12.48
*B. subtilis*	0.31	0.08	0.04	0.13
*B. cereus*	1.23	0.04	0.08	8.50

## Discussion

Actinomycins were firstly reported in 1940 from *Actinomyces antibioticus* (Waksman and Woodruff, 1940). Since then, more than 30 actinomycins have been discovered from the natural sources including Acts A_I_ (B_I_, X_I_), A_II_, A_IV_ (B_IV_, D, X_IV_), A_V_ (B_V_, X_V_), and Acts C_1_–C_3_ (Roussos and Vining, 1956), Acts E_1_, E_2_, and F_1_–F_4_ (Brockmann, 1961), Acts G_1_–G_6_ (Lackner et al., 2000a; Bitzer et al., 2006), X_0α_–X_0δ_ (Brockmann, 1961), Acts Y_1_–Y_5_ (Bitzer et al., 2009), and Acts Y_6_–Y_9_ (Cai et al., 2016), Acts Z_1_–Z_5_ (Lackner et al., 2000b), and Act Z_*p*_(Cai et al., 2016). To date, there are many *Streptomyces* species capable of producing actinomycins, but few of them were reported to produce relatively large quantities of one or two major actinomycins. *Streptomyces parvulus* (Foster and Katz, 1981), *S. halstedii, S. anulatus* (Praveen et al., 2008a), *S. sindenensis* (Praveen et al., 2008b), and *S. griseoruber* (Praveen and Tripathi, 2009) are examples to produce Act D, among which the highest production is 620 mg/L (Praveen et al., 2008a). *Streptomyces nasri* YG62 (Elnaggar et al., 1998) and *S. triostinicus* (Singh et al., 2009) are examples to produce Act X_2_ whose production reached to 443 mg/L (Singh et al., 2009). *Streptomyces* sp. MITKK-103 (Kurosawa et al., 2006), *Streptomyces* sp. JAU4234 (Xiong et al., 2008) and *Streptomyces* sp. MS449 (Chen et al., 2012) can simultaneously produce Acts D, X_0β_ and X_2_ and strain MS449 produced the highest production of Acts D and X_2_ with the yields of 1,770 and 1,920 mg/L, respectively. No examples were found to produce Act X_0β_ solely. This study identified Acts X_0β_, X_2_, and D by comparison of ^13^C NMR data to those reported ones (Lifferth et al., 1999; Wang et al., 2014) with the errors less than 0.5 ppm. And the productions of Acts D, X_0β_, and X_2_ by the marine-derived *S. heliomycini* WH1 in the optimized fermentation conditions were significantly improved by 100, 110, and 150 folds, respectively, relative to those in the isolation medium (MM medium). Among these actinomycins, Act-D has been extensively studied and widely used in the treatment of malignant tumors, such as Wilms' tumor and childhood rhabdomyosarcoma (Womer, 1997). However, the cytotoxicity of Acts X_2_ and X_0β_ against the tumor cells and antibacterial activities of Acts D, X_2_, and X_0β_ against pathogenic bacteria were received very little attention. Compared to Act D, Act X_2_ showed stronger cytotoxicity toward HL-60 cells (Kurosawa et al., 2006) and better antibacterial activity against MTB H37Rv (Chen et al., 2012) and *S. aureus* and *B. cereus* (Xiong et al., 2008). Our investigation firstly demonstrated that Act-X_2_ displayed the strongest activities against MCF-7, A549, and K562 human tumor cell lines, and the lowest toxicity on L02 human normal embryo liver cell line, indicating the potential use of Act-X_2_ as a cancer drug candidate. In addition, our fresh results on the strong antibacterial activity of Acts D and X_2_ against MRSA and *B. subtilis* along with Act-D against *B. cereus* indicated the potential use of Act-D and Act-X_2_ in the treatment of the infections caused by those human and aquatic pathogenic bacteria, especially by MRSA. Therefore, the present study revealed that actinobacteria from newly-explored, special or extreme environments could be a potential pool for drug discovery.

## Author contributions

The authors from China contribute to the isolation and identification of Acts, the optimization of fermentation conditions, as well as the assays of the cytotoxic and antimicrobial activities, and prepared the paper. The authors from Saudi Arabia are responsible for the isolation and identification of the marine-derived actinobacterial strain, *Streptomyces heliomycini* WH1.

### Conflict of interest statement

The authors declare that the research was conducted in the absence of any commercial or financial relationships that could be construed as a potential conflict of interest.

## Data sheet

The colony and micrograph of *Streptomyces heliomycini* WH1 and the MS and NMR spectra of compounds **1**–**3**.
