# Supplementary material for: Identification, Bioactivity, and Productivity of Actinomycins from the Marine-Derived Streptomyces heliomycini
Source: Front Microbiol. 2017 Jun 28;8:1147. doi: 10.3389/fmicb.2017.01147 (PMC5487404; doi:10.3389/fmicb.2017.01147)

Identification, Bioactivity and Productivity of Actinomycins from the Marine-Derived *Streptomyces heliomycini*

**Dongyang Wang^1^•Cong Wang^1^•Pengyan Gui^1^•Haishan Liu^1^•Sameh M.H. Khalaf ^2^•Elsayed A. Elsayed^2^• Mohammed A.M. Wadaan^2^•Wael N. Hozzein^2, *^•Weiming Zhu^1, *^**

**^1^** Key Laboratory of Marine Drugs, MEC, School of Medicine and Pharmacy, Ocean University of China, Qingdao 266003; **^2^** Bioproducts Research Chair, Zoology Department, College of Science, King Saud University, Riyadh 11451, Kingdom of Saudi Arabia.

* To whom should be correspondence, E-mail: weimingzhu@ouc.edu.cn (W. Zhu); hozzein29@yahoo.com (W. Hozzein);

**Table of contents**

______________________________________________________________________

**The physicochemical properties of Acts X_0β_, X_2_ and D**……………………………………….…S2

**Table S1** The productions of Acts X_0β_, X_2_ and D in MM medium with different initial pH…………..S3

**Table S2** The productions of Acts X_0β_, X_2_ and D in MM medium with different salinity………….. S3

**Table S3** The productions of Acts X_0β_, X_2_ and D in MII medium with different initial pH………….S3

**Table S4** The productions of Acts X_0β_, X_2_ and D in MII medium with different salinity……………...S3

**Fig. S1**. The electron micrograph of strain *Streptomyces heliomycini* WH1 …………………………S4

**Fig. S2**. The colony of *Streptomyces heliomycini* WH1 ……………………………………………...S4

**Fig. S3**. HPLC profiles of the cultures of *Streptomyces* *heliomycini* WH1…………………………S4

**Fig. S4**. The ^1^H NMR (600 MHz, CDCl_3_) spectrum of Act-X_0β_……………………..…..……………S5

**Fig. S5**. The ^13^C NMR (150 MHz, CDCl_3_) spectrum of Act-X_0β_………………………….…………S5

**Fig. S6**. The ESI-MS of Act-X_0β_………………………………......…………………….……............S6

**Fig. S7**. The ^1^H NMR (500 MHz, CDCl_3_) spectrum of Act-X_2_…………………………….…………S6

**Fig. S8**. The ^13^C NMR (125 MHz, CDCl_3_) spectrum of Act-X_2_……………..………………………S7

**Fig. S9**. The ESI-MS of Act-X_2_…………………………………………................…………………S7

**Fig. S10**. The ^1^H NMR (500 MHz, CDCl_3_) spectrum of Act-D…………………….……………….S8

**Fig. S11**. The ^13^C NMR (125 MHz, CDCl_3_) spectrum of Act-D………………..………….…………S8

**Fig. S12**. The ESI-MS of Act-D…………………………………………………..………………..…S9

_________________________________________________________________

**The physicochemical properties of Acts X_0β_, X_2_ and D**

Act-X_0β_ (**1**): amorphous powder. [α]_D_^25^ –53 (*c* 0.2, MeOH); UV (MeOH) λ_max_ (log *ε*) 214 (3.00), 222 (3.34) and 442 (1.79) nm; IR (KBr) *ν*_max_ 3304, 1748, 1643, 1512, 1489, 1300, 1194, 1028 cm^-1^; ^1^H and ^13^C NMR data, see Table 1, Fig. S4 and S5; ESIMS *m/z* 1272.06[M+H]^+^, 1294.05[M+Na]^+^, HRESIMS m/z 1271.6305 [M+H]^+^ (calcd for C_62_H_87_O_17_N_12_, 1271.6307) (Fig. S6).

Act-X_2_ (**2**): amorphous powder. [α]_D_^25^ –268 (*c* 0.2, MeOH); UV (MeOH) λ_max_ (log *ε*) 214 (2.62), 222 (2.83) and 442 (1.83) nm; IR (KBr) *ν*_max_1750, 1643, 1583, 1511, 1487, 1300, 1193 cm^-1^; ^1^H and ^13^C NMR data, see Table 2, Fig. S7 and S8; ESIMS *m/z* 1270.03[M+H]^+^, 1292.03[M+Na]^+^ , HRESIMS m/z 1269.6155 [M+H]^+^ (calcd for C_62_H_85_O_17_N_12_, 1269.6150) (Fig. S9).

Act-D (**3**): amorphous powder. [α]_D_^25^ –210 (*c* 0.2, MeOH); UV (MeOH) λ_max_ (log *ε*) 214(2.59), 222 (2.65) and 442 (1.99) nm; IR (KBr) *ν*_max_3289, 1747, 1643, 1582, 1511, 1486, 1383, 1193, 1127 cm^-1^; ^1^H and ^13^C NMR data, see Table 3, Fig. S10 and S11; ESIMS *m/z* 1256.05[M+H]^+^, 1278.06[M+Na]^+^, 1294.04[M+K]^+^, HRESIMS m/z 1255.6365 [M+H]^+^ (calcd for C_62_H_87_O_16_N_12_, 1255.6358) (Fig. S12).

**Table S1**. The productions of Acts X_0β_, X_2_ and D in MM medium with different initial pH (mg/L)

| Initial pH | Act X_0β_ | Act X_2_ | Act D |
| --- | --- | --- | --- |
| 4.5 | 0.31 | 1.79 | 0.94 |
| 5.0 | 0.54 | 3.48 | 1.87 |
| 5.5 | 0.96 | 6.51 | 3.69 |
| 6.0 | 1.15 | 4.71 | 3.39 |
| 6.5 | 0.90 | 4.01 | 3.42 |
| 7.0 | 0.79 | 3.39 | 2.88 |
| 7.5 | 0.61 | 2.61 | 3.37 |
| 8.0 | 0.50 | 3.27 | 2.96 |
| 8.5 | 0.48 | 2.83 | 3.26 |
| 9.0 | 0.08 | 0.55 | 0.28 |

**Table S2**. The productions of Acts X_0β_, X_2_ and D in MM medium with different salinity (mg/L)

| NaCl | Act X_0β_ | Act X_2_ | Act D |
| --- | --- | --- | --- |
| 0% | 0.00 | 0.31 | 0.01 |
| 3% | 0.52 | 2.11 | 2.21 |
| 5% | 2.50 | 7.34 | 6.80 |
| 7% | 0.04 | 0.08 | 0.26 |
| 9% | 0.00^a^ | 0.00^a^ | 0.00^a^ |

^a^ strains didn’t grow in this condition

**Table S3**. The productions of Acts X_0β_, X_2_ and D in MII medium with different initial pH (mg/L)

| Initial pH | Act X_0β_ | Act X_2_ | Act D |
| --- | --- | --- | --- |
| 5.0 | 0.00^a^ | 0.00^a^ | 0.00^a^ |
| 5.5 | 14.49±0.99 | 34.09±8.76 | 64.75±6.42 |
| 6.0 | 9.05±1.31 | 24.59±4.67 | 45.40±11.12 |
| 6.5 | 12.58±0.78 | 32.67±4.36 | 78.61±6.20 |
| 7.0 | 37.20±4.61 | 82.40±4.20 | 386.49±56.99 |
| 7.5 | 53.79±1.11 | 115.02±11.61 | 454.03±59.90 |
| 8.0 | 56.81±6.82 | 112.43±27.21 | 428.54±34.55 |
| 8.5 | 68.75±1.20 | 145.70±6.85 | 456.53±14.74 |
| 9.0 | 50.35±7.70 | 83.23±8.45 | 278.53±7.74 |
| 9.5 | 0.00^a^ | 0.00^a^ | 0.00^a^ |

^a^ strains didn’t grow in this condition

**Table S4**. The productions of Acts X_0β_, X_2_ and D in MII medium with different salinity (mg/L)

| NaCl | Act X_0β_ | Act X_2_ | Act D |
| --- | --- | --- | --- |
| 0% | 26.39±7.17 | 77.07±16.38 | 37.27±8.84 |
| 3% | 68.75±1.20 | 145.70±6.85 | 456.53±14.74 |
| 5% | 107.64±4.22 | 283.44±75.30 | 458.00±76.34 |
| 7% | 50.56±6.02 | 102.18±16.93 | 200.60±23.82 |
| 9% | 0.00^a^ | 0.00^a^ | 0.00^a^ |

^a^ strains didn’t grow in this condition

**Fig.** **S1**. The electron micrograph of strain *Streptomyces heliomycini* WH1 ^a^.


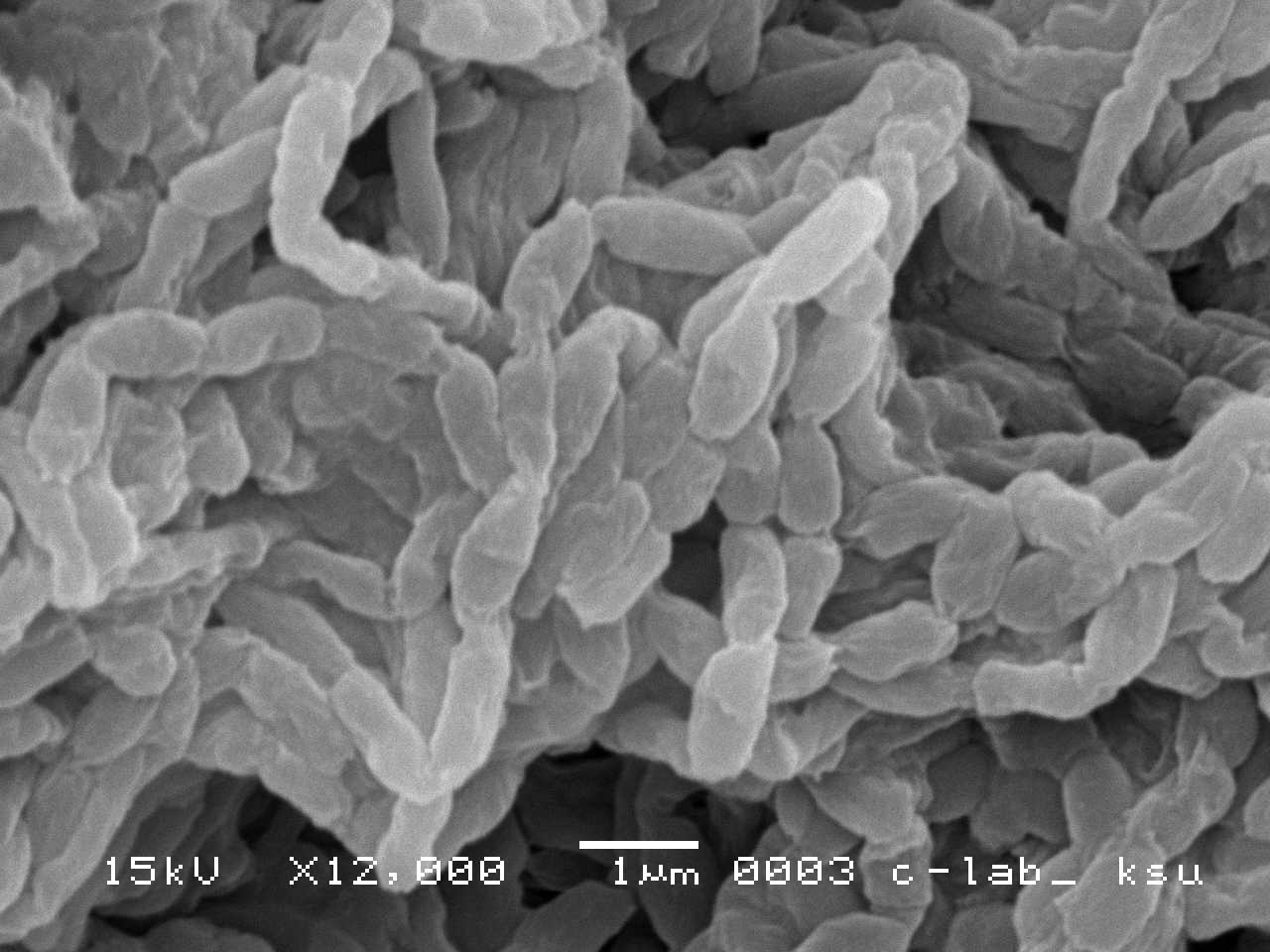


^a^ showing the long spore chains with the smooth surfaces

**Fig.** **S2**. The colony of *Streptomyces heliomycini* WH1 ^a^.


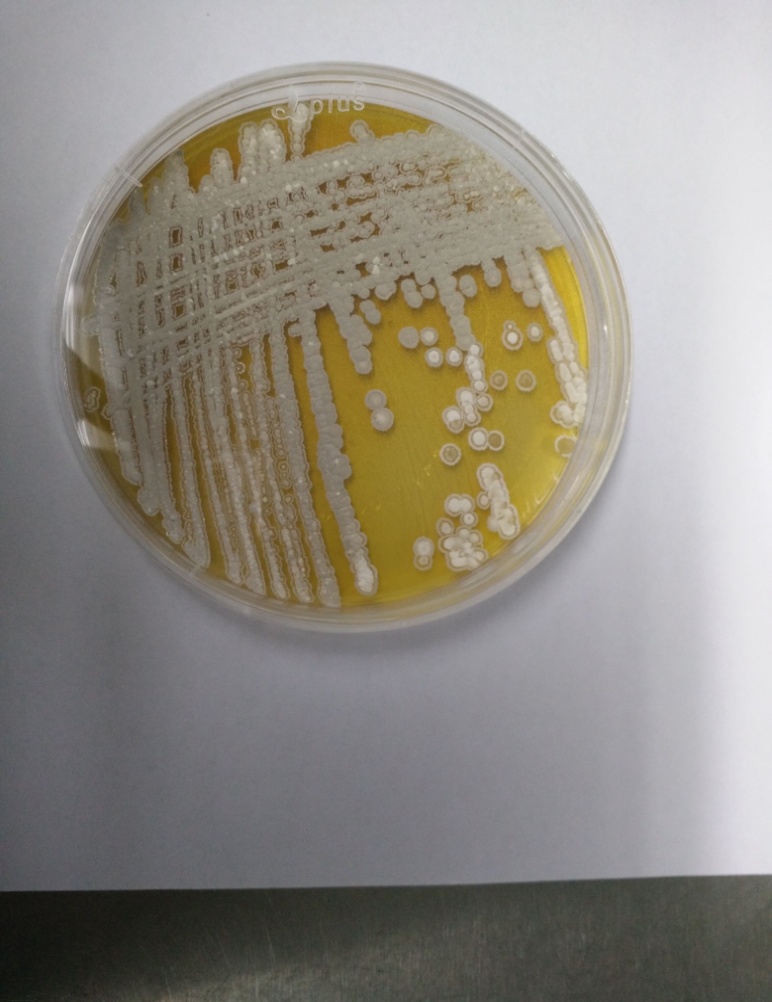


^a^ growth for 10-d on medium ISP2 at 28˚C

**Fig. S3**. HPLC profiles of the cultures of *Streptomyces heliomycini* WH1 ^a^.


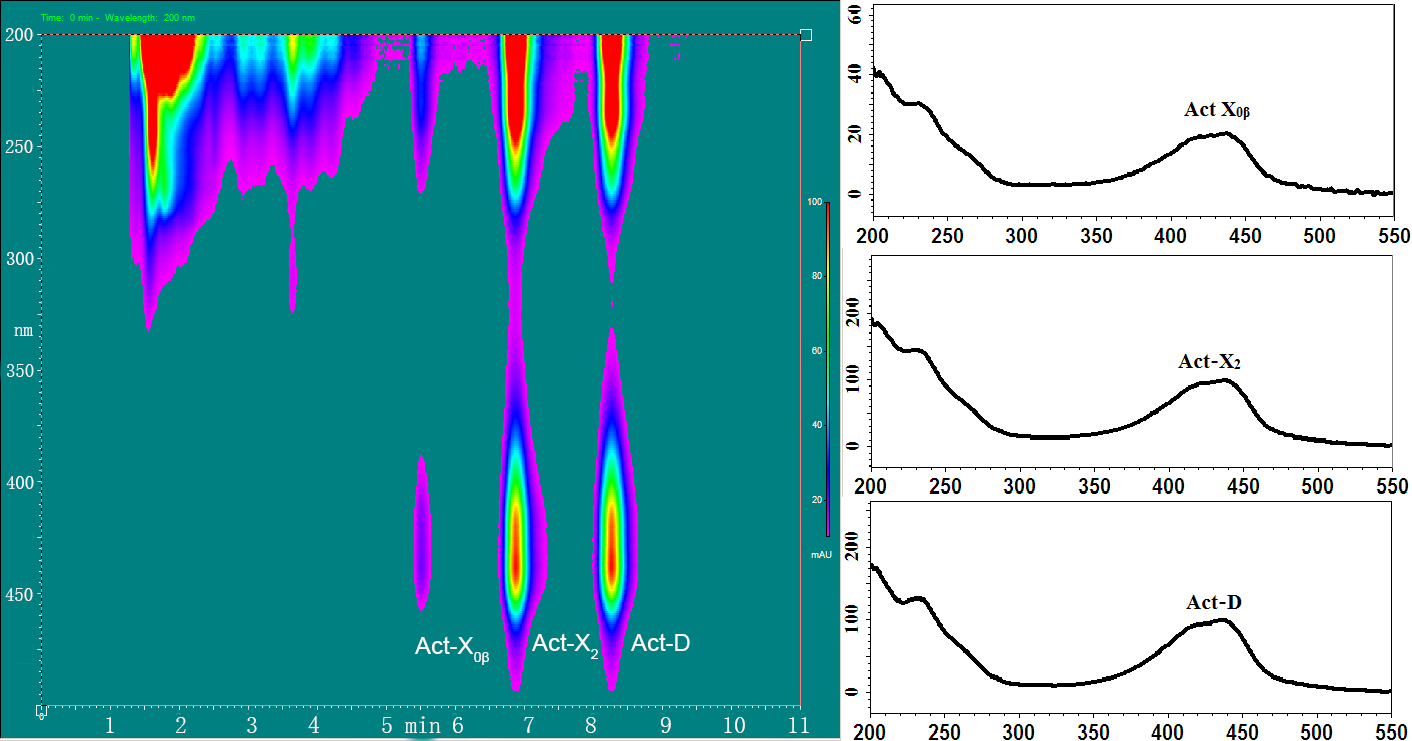


^a^ analysis by RP-8 column eluting with 80% MeOH-H_2_O (v/v)

**Fig. S4**. The ^1^H NMR (600 MHz, CDCl_3_) spectrum of Act-X_0β_


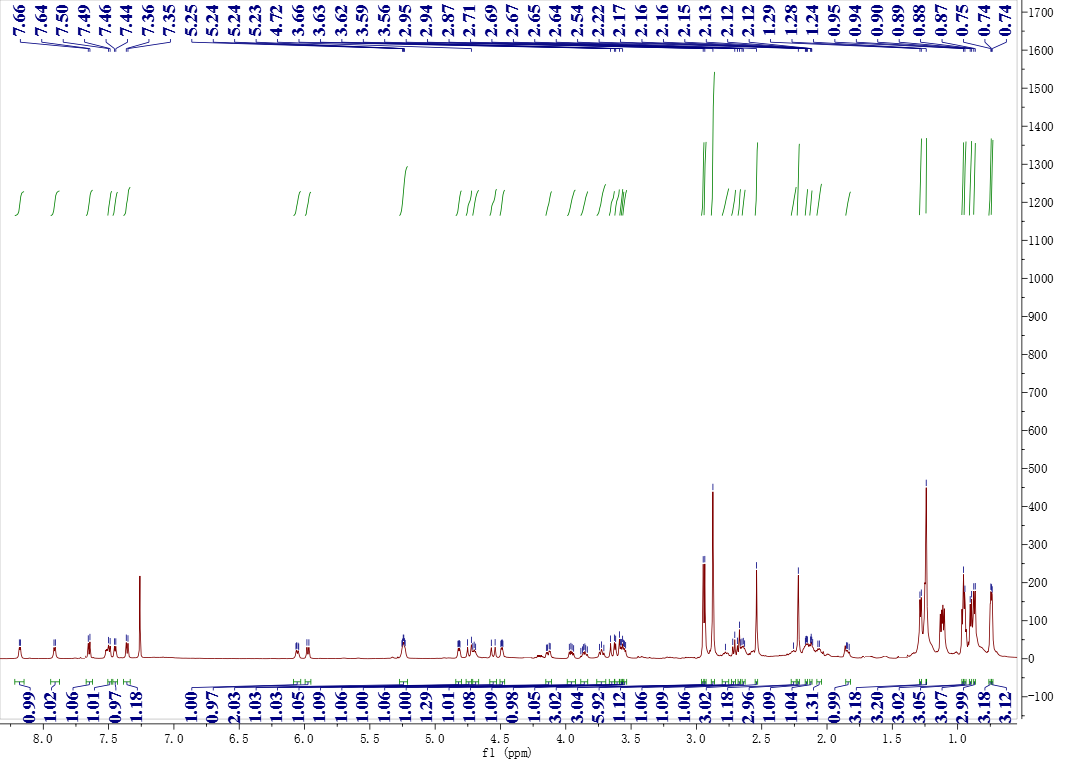


**Fig. S5**. The ^13^C NMR (150 MHz, CDCl_3_) spectrum of Act-X_0β_

**Fig. S6**. The ESI-MS of Act-X_0β_


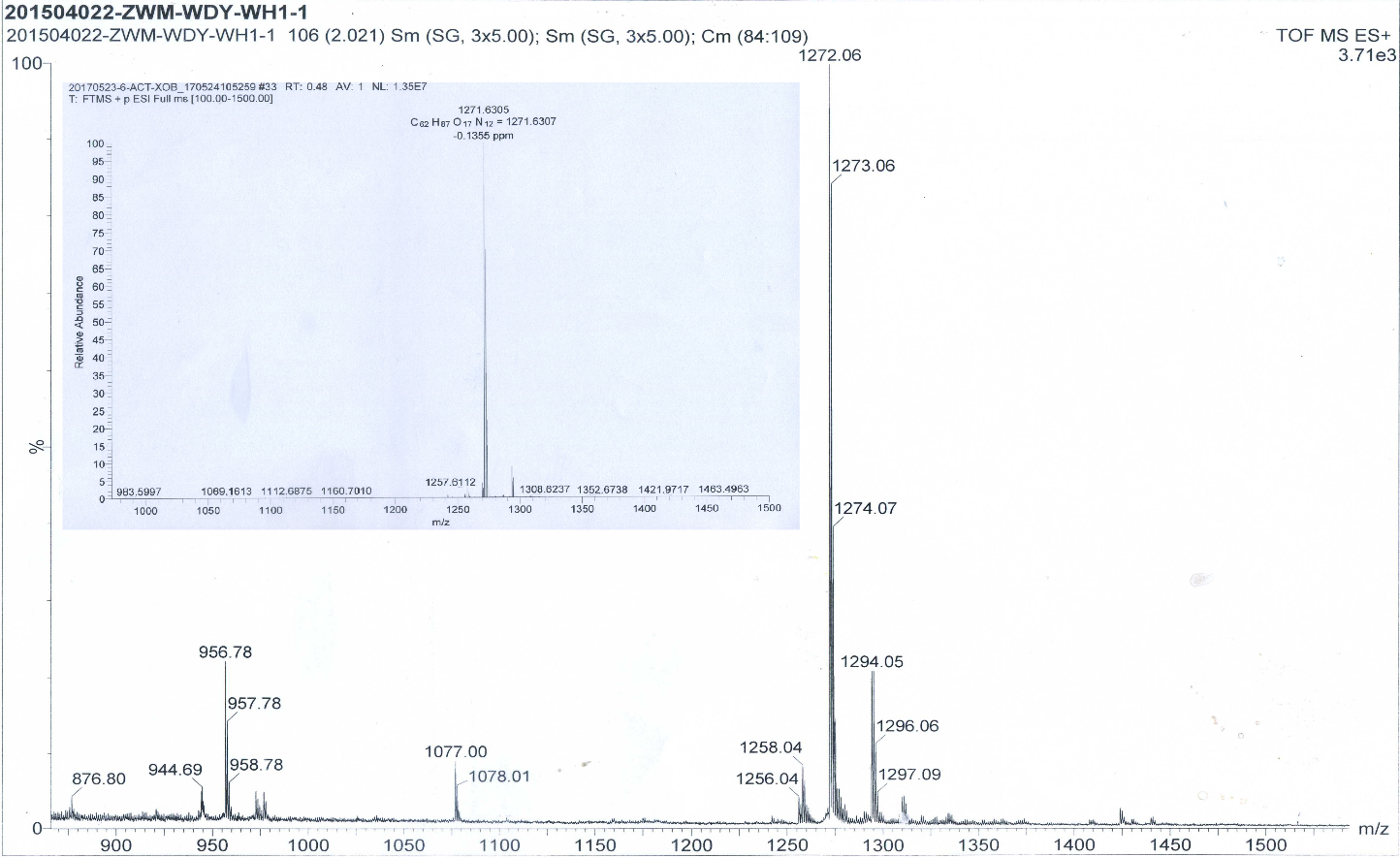


**Fig. S7**. The ^1^H NMR (500 MHz, CDCl_3_) spectrum of Act-X_2_

**Fig. S8**. The ^13^C NMR (125 MHz, CDCl_3_) spectrum of Act-X_2_

**Fig. S9**. The ESI-MS of Act-X_2_


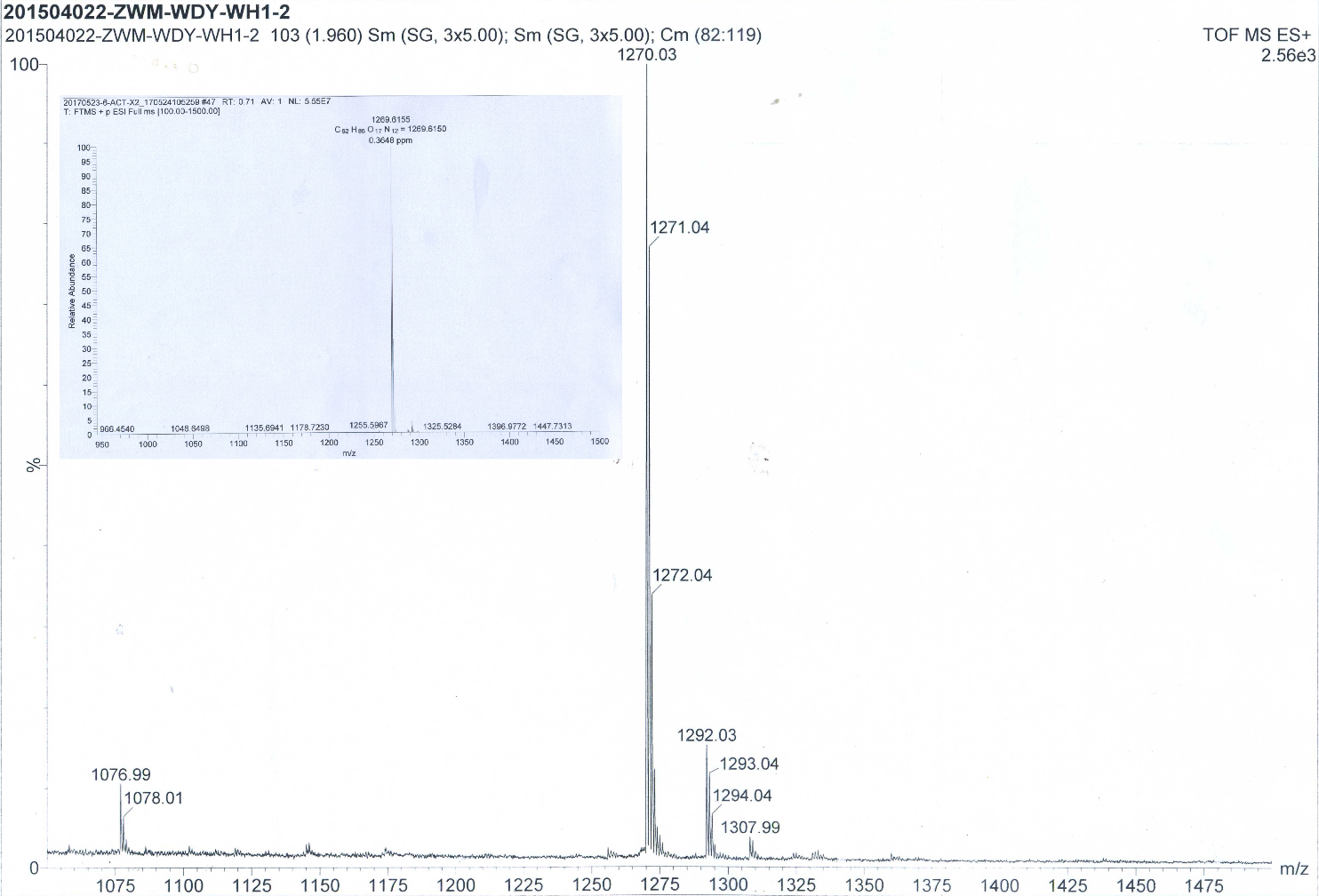


**Fig. S10**. The ^1^H NMR (500 MHz, CDCl_3_) spectrum of Act-D

**Fig. S11**. The ^13^C NMR (125 MHz, CDCl_3_) spectrum of Act-D


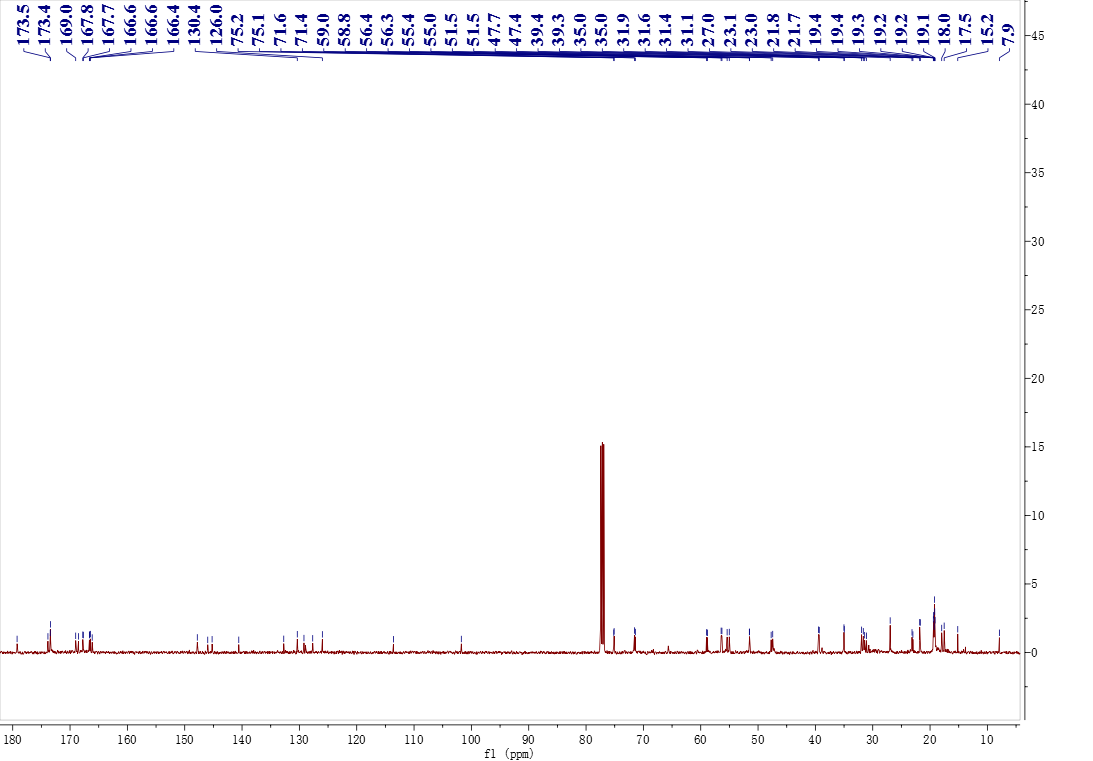


**Fig. S12**. The ESI-MS of Act-D


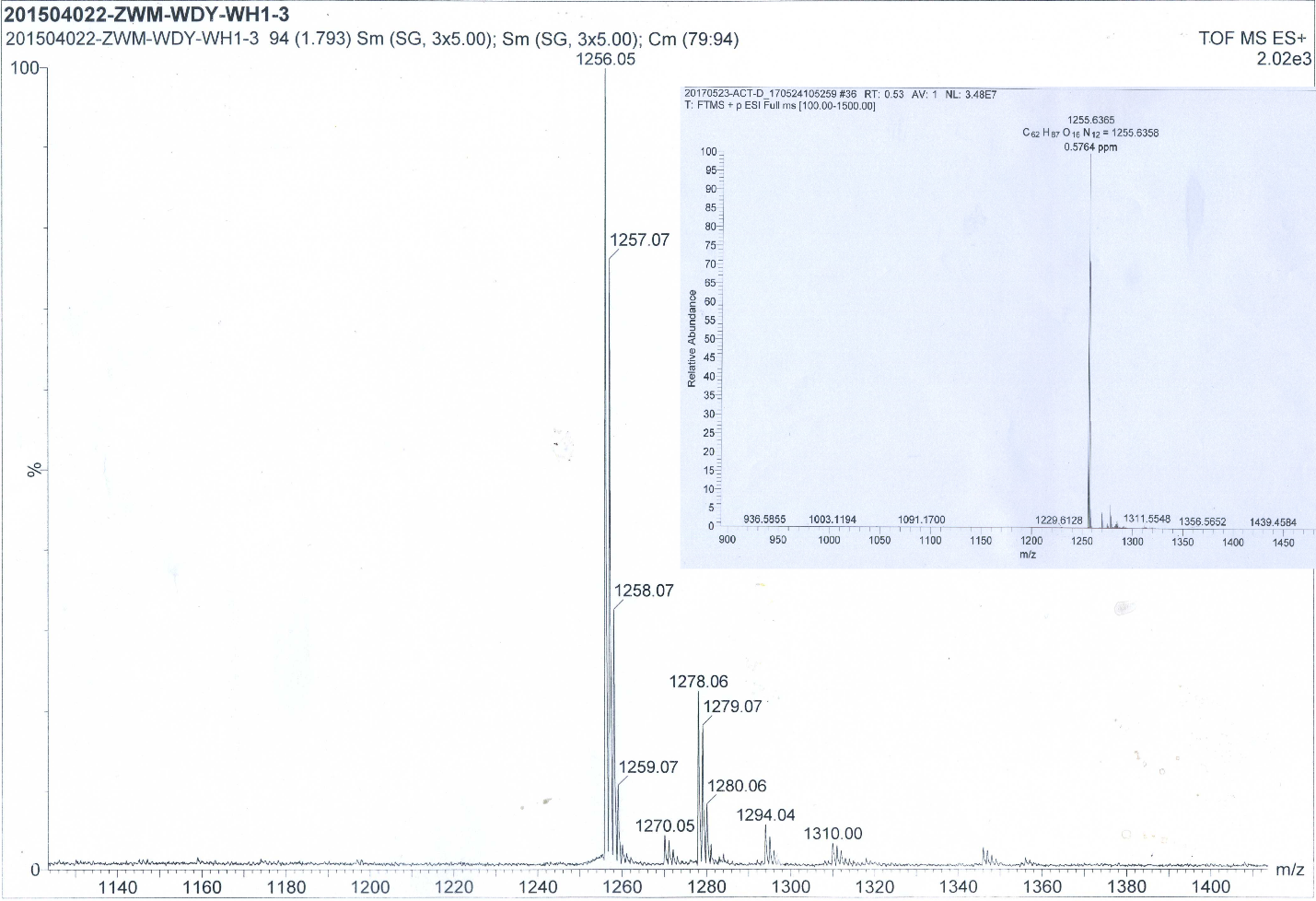

Supplement: Supplementary file 1 [file DataSheet1.docx]
